# Method for diagnosing neoplastic lesions by quantitative fluorescence value

**DOI:** 10.1038/s41598-019-44287-z

**Published:** 2019-05-24

**Authors:** Ayaka Kosugi, Masataka Kasahara, Longqiang Yang, Aki Nakamura-Takahashi, Takahiko Shibahara, Taisuke Mori

**Affiliations:** 1 Department of Pathology and Clinical Laboratories, National Cancer Center Hospital, Tokyo, Japan; 2 Department of Oral Maxillofacial Surgery, Tokyo Dental College, Tokyo, Japan; 3 Department of Pharmacology, Tokyo Dental College, Tokyo, Japan

**Keywords:** Oncogenes, Oral cancer detection

## Abstract

Fluorescence visualization devices (FVs) are useful for detecting malignant lesions because of their simple and noninvasive application. However, their quantitative application has been challenging. This study aimed to quantitatively and statistically evaluate the change in fluorescence intensity (FI) during the progression from normal epithelium to squamous cell carcinoma using a reproducible animal tongue carcinogenesis model. To establish this model, rats were treated with 50 ppm 4-Nitroquinoline 1-oxide (4NQO) in their drinking water for 10, 15, and 20 weeks. After 4NQO administration, each rat tongue was evaluated by gross observation, histology, and FI measurements. Fluorescence images were captured by FV, and ImageJ was used to measure FI, which was analyzed quantitatively and statistically. The establishment of a reproducible tumor progression model was confirmed, showing precancerous lesions (low-grade dysplasia [LGD]), early cancers (high-grade dysplasia/carcinoma *in situ* [HGD/CIS]), and advanced cancers (Cancer). This carcinogenesis model was quantitatively evaluated by FI. The FI of LGD stage was 54.6, which was highest intensity of all groups. Subsequently, the HGD/CIS and Cancer stages showed decreased FI (HGD/CIS: 46.1, Cancer: 49.1) and manifested as dark spots. This result indicates that FI had more variation and a wider range with increasing tumor progression. We demonstrated that FI migration and an uneven distribution are consistent with tumor progression. Since each step of tumor progression occurs reproducibly in this animal model, statistical evaluation was possible. In addition, tumor progression can be monitored by this new FI analysis method in humans.

## Introduction

The morbidity of oral cancer has increased over the past few decades. While treatment methods are improving, survival rates remain low, largely because it is hard to distinguish oral mucosal lesions, such as oral potentially malignant disorders (OPMDs), from early stage cancer. Although the oral cavity can be diagnosed via direct viewing, the early detection of oral premalignant or cancerous lesions remains difficult^1^. The grade of dysplasia is not easy to differentiate by general pathologists. If these lesions have to be monitored over time, the use of non-invasive methods rather than repeated biopsies will benefit patients. Therefore, noninvasive methods are preferable for diagnosing these lesions at an early stage.

Recently, it has been demonstrated that fluorescence visualization devices (FVs) are useful for detecting lesions of the oral mucosa, and especially to discriminate between OPMDs such as oral lichen planus, leukoplakia, and dysplasia^2–6^. Among its benefits, the FV system is simple and noninvasive to use^7,8^. The IllumiScan® FV (Shofu Inc, Kyoto, Japan) uses 425 nm wavelength visual light to distinguish between normal and abnormal mucosa^9–11^. Normal oral mucosa has a fluorescence spectral range of approximately 375 nm to 440 nm. Under this light, normal mucosa emits pale green fluorescence while abnormal areas absorb the fluorescent light, resulting in dark patches with fluorescence visualization loss (FVL)^12,13^. However, it has not yet been determined why abnormal parts of the mucosa cause FVL. It has been shown that FV can detect differences in fluorescence between normal mucosa and epithelial dysplasia^1,3,14^. Other reports have demonstrated that FV can be used as a diagnostic tool of for oral malignant disorders, but such observations have not been evaluated quantitatively^15^. This lack of quantification may be a result of human oral cancer being caused by many factors, including smoking, alcohol consumption, and viral infection. Moreover, different parts of the oral cavity mucosa vary in the degree of keratinization and thickness. Another issue with FV is that examinations are highly subjective, depending strongly on the experience of the examiner, and can be complicated by inflammation or reflection from surrounding materials^16,17^. Therefore, evaluations are not consistent, and there is little evidence to quantitatively evaluate oral lesions in mucosal tissue that is not clearly normal or malignant.

**Figure 1:**
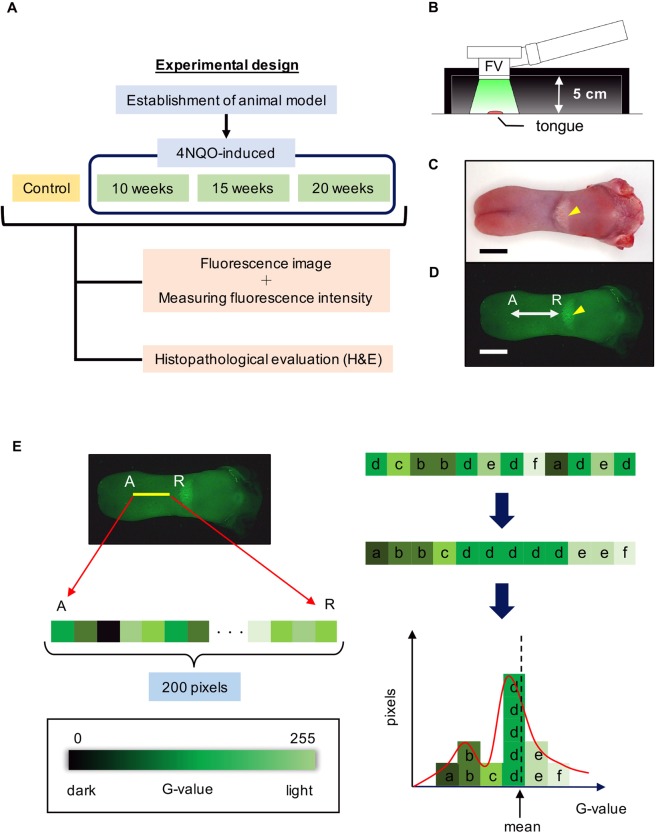
Experimental design for fluorescence visualization. Flow chart of the experimental design. (**A**) Method for recording fluorescence images. (**B**) The focal length of the camera to the floor was 5 cm. Gross observation (**C**) and fluorescence images (**D**) of a normal rat tongue are shown. The tongue contains a “half-moon structure” (yellow arrow head). The measuring sight of the anterior tongue corresponded to 200 pixels (white arrow, A: tongue apex, R: tongue root, scale bar: 10 mm).

To overcome this problem, we examined the transition of disease from normal tissue to low grade dysplasia (LGD), high grade dysplasia (HGD), carcinoma *in situ* (CIS), and squamous cell carcinoma (Cancer)^18^, using a reproducible 4-nitroquinoline 1-oxide (4NQO)-induced rat cancer model^19,20^. We also statistically evaluated the relationship between tumor progression and fluorescence pattern using the “G-value” intensity unit. The aim of this study was to quantitatively evaluate the early stages of cancer by a new, noninvasive FV-based analysis method. Here, we demonstrate this evaluation system for accurately digitized observations by using a reproducible cancer model under consistent conditions (Fig. 1).

## Results

**Figure 2:**
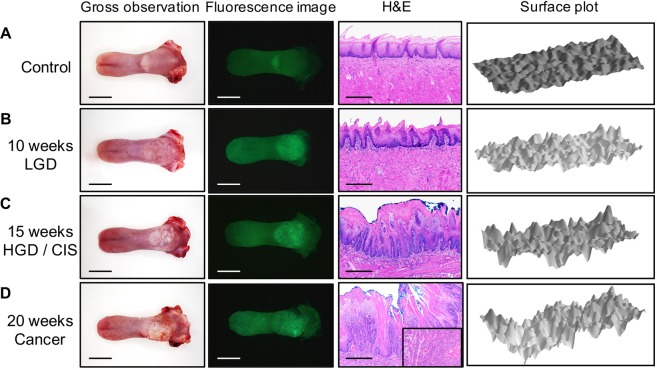
Accurate reproduction of tongue carcinogenesis. Each panel shows four images: gross observation, fluorescence image, hematoxylin and eosin (H&E) stain, and surface plot for a control tongue (**A**), low-grade dysplasia (**B**), high-grade dysplasia/carcinoma *in situ* (**C**), and cancer (**D**). For the gross observation and fluorescence images, scale bars indicate 10 mm. For H&E staining, the magnification was ×100, and scale bars indicate 250 μm. Surface plots show three-dimensional graphs of fluorescence intensities.

We established a reproducible tumor progression model of precancerous lesions (LGD), early cancers (HGD/CIS) and advanced cancers (Cancer) from normal tongues. Approximately uniform tumor progression was observed in the samples within each group. The control group showed normal gross findings and emitted slightly darkened green fluorescence, except for the half-moon structure. Histopathology revealed normal findings, and the surface plot was smooth with a uniform height (Fig. 2A). In contrast, in the group treated with 4NQO for 10 weeks, gross observation of the epithelial mucosa revealed a rough surface with erythema. The whole tongue mucosa emitted strong green fluorescence, and histopathology findings were consistent with LGD. The LGD surface plot showed that the tongue had become bumpy and exhibited height differences (Fig. 2B). In the group treated with 4NQO for 15 weeks, the epithelial mucosa appeared to have become rougher, appearing as a granular mucosal surface with varying degrees of erythema. The fluorescence image revealed irregular dark and light areas across the whole tongue. The whole tongue mucosa emitted green fluorescence, but the anterior half-moon structure was darker than in the LGD stage, and the area of the posterior half-moon structure was larger. Histopathology corresponded to HGD and CIS. The HGD/CIS surface plot showed more roughness than that of LGD, and the fluorescence image showed an increase in the dark fluorescent area (Fig. 2C). In the group treated with 4NQO for 20 weeks, the epithelial mucosa was very rough, exhibiting hyperkeratosis and a granular mucosal surface with varying degrees of erythema across the whole tongue. The fluorescence image showed pale green fluorescence and multiple irregular dark spots. Histopathology indicated invasive squamous cell carcinoma (Cancer). The surface plot of the Cancer group showed height differences and the most roughness of all groups. Moreover, there were many scattered dark spots across the surface (Fig. 2D).

### Quantitative analysis of fluorescence intensity during tumor progression

**Figure 3:**
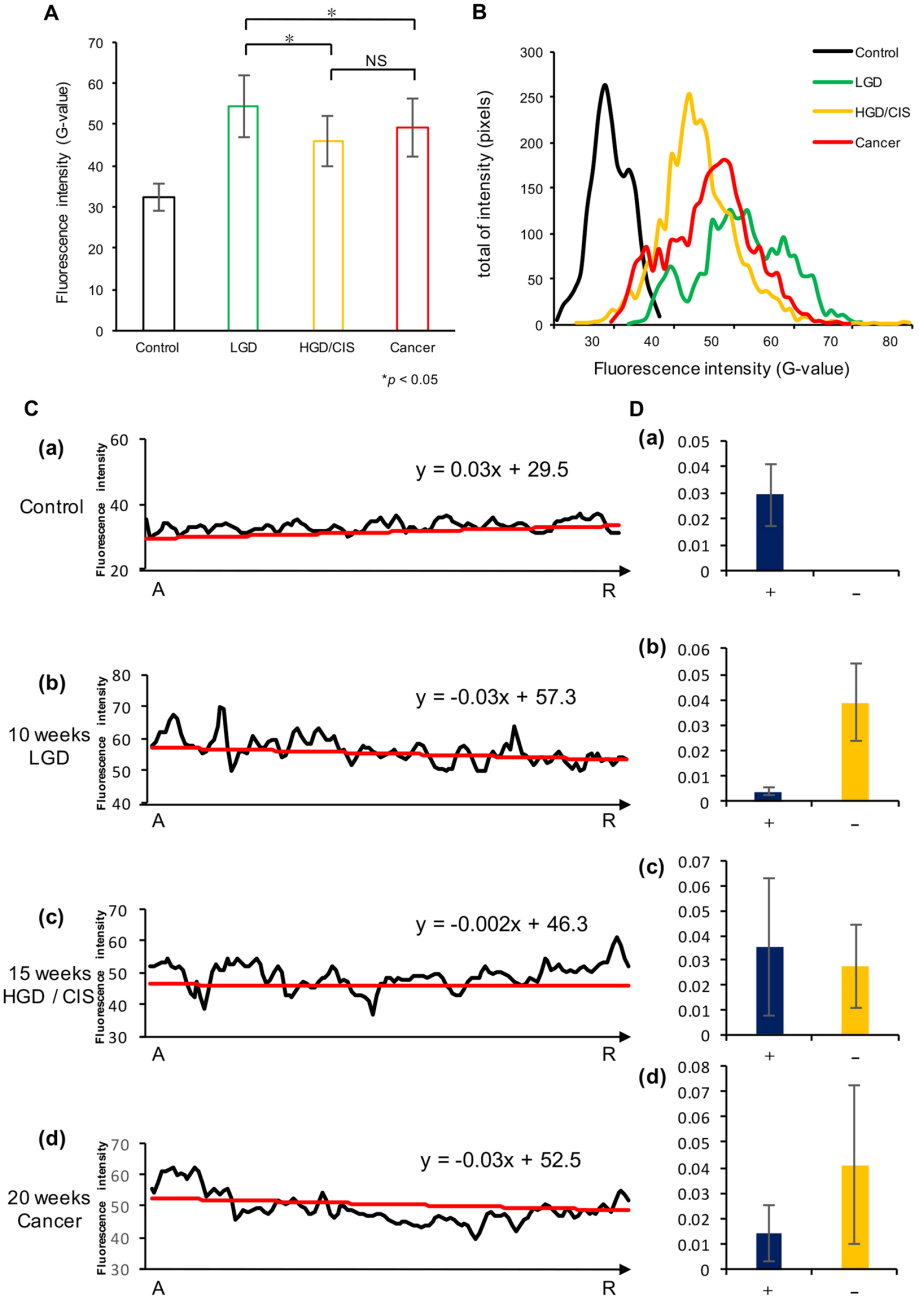
The analysis of the intensity elements. (**A**) Graphs show the mean fluorescence intensity of each group. The vertical axis shows fluorescence intensity (G-value). All experimental groups had significantly higher intensity than Control, while the intensity of high-grade dysplasia/carcinoma *in situ* (HGD/CIS) and Cancer was significantly darker than that of low-grade dysplasia (LGD). However, HGD/CIS was not significantly darker than cancer (p < 0.05, Dunnett’s test). (**B**) Changes in fluorescence intensity and distribution between the groups. The pixels showing fluorescence were plotted to examine their distribution. (**C**) Representative example of fluorescence measurements at each pixel (black line) and the mean inclination line (y = ax + b, red line). The inclination line indicated the minimum distance from the measured intensity for all pixels. The horizontal axis indicates the distance from the tongue apex (A) to tongue root (R), while the vertical axis shows the fluorescence intensity (G-value). (**D**) The corresponding bar graph shows the average slopes of the linear approximations, separated by whether they showed positive (+) or negative (−) slopes. Error bars indicate standard deviation.

Figure 3A shows the FI (quantified as G-values) in all four groups as vertical bars. The FI of the control group was 32.5 ± 3.2, and it changed to 54.6 ± 7.5 for LGD. Subsequently, the FI decreased to 46.1 ± 6.2 in HGD/CIS. Finally, in the Cancer stage, the average FI was 49.1 ± 7.1. The control group had significantly different FI from the other three groups. While the HGD/CIS and Cancer groups were not significantly different from each other (p < 0.05, Dunnett’s test), they had significantly lower FI than the LGD group. These results showed that FI significantly differs between normal and abnormal epithelium with malignant changes, allowing lesions of malignant grade to be discriminated from dysplasia. In addition, we determined whether the degree of tumor progression was uniform within each group. All groups demonstrated a high concordance rate (p < 0.01, Pearson Correlation) and tumor progression in the 4NQO-treated groups had same level of malignancy (Table 1).

**Table 1:** Quantitative analysis of fluorescence intensity and comparison of each distribution.

	n	Mean	SD	Median	Min	Max	Range	Mean of PCC
								(lower to higher)
Control	10	32.5	3.2	32.2	23.6	41	17.4	0.89
								(0.80–0.98)
LGD	11	54.6	7.5	54.5	35.7	74.8	39.1	0.84
								(0.70–0.97)
HGD/CIS	15	46.1	6.2	45.6	25.3	81.4	56.1	0.89
								(0.70–0.98)
Cancer	14	49.1	7.1	49.6	32.1	72.3	40.2	0.85
								(0.60–0.98)
PCC: Pearson correlation coefficient.								*p* < 0.01

### Scattering and distribution of fluorescence intensity

Next, we evaluated the range of FI in each group and its distribution between all groups. In Fig. 3B, the horizontal axis shows FI (G-value) and the vertical axis shows the total FI of all measured pixels. The control group showed a median G-value of 32.2, and the FI spanned a narrow range of approximately 25 to 35. The control group exhibited hardly any scattering. In LGD group, the FI showed a median G-value of 54.5 and was shifted to a generally high intensity. The FI values were widely distributed, with a G-value range of approximately 35 to 75. In the HGD/CIS group, the median G-value decreased to 45.6, and the overall fluorescence intensity distribution was shifted to low levels. The G-value range was approximately 25 to 80, which was the widest among the groups. Finally, progressing to the Cancer stage, the median FI was a G-value of 49.6, with values spanning a large range of approximately 30 to 70. The fluorescence distribution of the LGD to Cancer stages markedly differed from that of the control group. Thus, differences in tumor progression can be distinguished based on the extent of FI distribution (Table 1).

Furthermore, FI was evaluated as a cut off threshold at each stage. Control versus lesions indicated that the mean sensitivity and specificity were 94% and 97.5%, respectively (Table 2 # 1, 2, 3 and 4). These comparisons were 38.6 as the average cut off of FI values. LGD versus HGD and cancer group the sensitivity was above 70% and the average cut off G value was 50.8 (Table 2 # 5, 6 and 10). In other comparisons including at the cancer stage, specificity was not high, so clear FI threshold could not be shown. As with the human case, we evaluated the FI as threshold in each of the four stages. The result showed Control vs HGD was 96% as sensitivity and 98% as specificity, and the cut off average of G-value was 39.4 (Table 2 #2).

**Table 2:** ROC Curve to confirm G-value Cutoff Threshold.

Animal model	Cut off (G-value)	AUC	*p*	Sensitivity (%)	Specificity (%)
1. Control vs LGD, HGD/CIS, Ca	38.7	0.99	<0.001*	93	98
2. Control vs LGD	39.4	0.99	<0.001*	99	99
3. Control vs HGD	38.5	0.98	<0.001*	91	97
4. Control vs Ca	37.8	0.99	<0.001*	93	96
5. LGD vs HGD	48.7	0.81	<0.001*	71	80
6. LGD vs Ca	52.7	0.7	<0.001*	70	60
7. HGD vs Ca	48.5	0.64	<0.001*	57	70
8. Control, LGD vs HGD, Ca	37.4	0.6	<0.001*	94	45
9. Control, LGD, HGD vs Ca	46.3	0.63	<0.001*	68	56
10. LGD vs HGD, Ca	50.9	0.76	<0.001*	70	69
11. LGD, HGD vs Ca	60.8	0.5	<0.023*	96	11
**Human**	**Cut off (G-value)**	**AUC**	***p***	**Sensitivity (%)**	**Specificity (%)**
1. Control vs LGD	40.4	0.71	<0.001*	54	100
2. Control vs HGD	39.4	0.97	<0.001*	96	98
3. Control vs Ca	32.9	0.58	0.4195	38	100
4. LGD vs HGD	39.4	0.74	<0.001*	98	45
5. LGD vs Ca	36.8	0.68	<0.0074*	56	80
6. HGD vs Ca	38.5	0.8	<0.001*	68	96

### Pattern of varying fluorescence intensity

In Fig. 3C, which presents data from a single representative sample, the black line indicates the FI (vertical axis) from the tongue apex (A) to root (R), while the calculated inclination line is shown in red. The corresponding bar graph (Fig. 3D) accumulates whether it has a positive (+) or negative (−) gradient from the value of the approximate straight line. For the control group, FI showed little variation across the tongue, and the slope of the line had a positive value of 0.03. There was little scattering of intensity. This slopes of the control group were all positive in value and had a small standard deviation. Therefore, the control slopes did not exhibit scattering of intensity (Fig. 3C,D-a). The graph for the LGD group showed larger variance than that of the control group, and the scattering of the pixels was increased. The mean slope for LGD was −0.03, and most slopes were negative but with a large standard deviation. Thus, the scattering in the LGD group was increased compared to the control (Fig. 3C,D-b). The HGD/CIS group showed more variation than the LGD group, with a mean slope of 0.002, derived from an average of 0.04 for the positive values and −0.03 for the negative values (Fig. 3C,D-c). The graph of the cancer group showed the widest variability and the slope of the mean line was −0.03, from an average of 0.01 for the positive values and −0.04 for the negative values (Fig. 3C,D-d). Notably, the negative values exhibited a large degree of variation in slopes. This result indicates that FI had more variation and a wider range with increasing tumor progression. Furthermore, the three experimental groups showed a negative slope, which was consistent with a phenomenon in which the luminance declined in the central part of the lesion.

### Practical application for human tongue cancer

**Figure 4:**
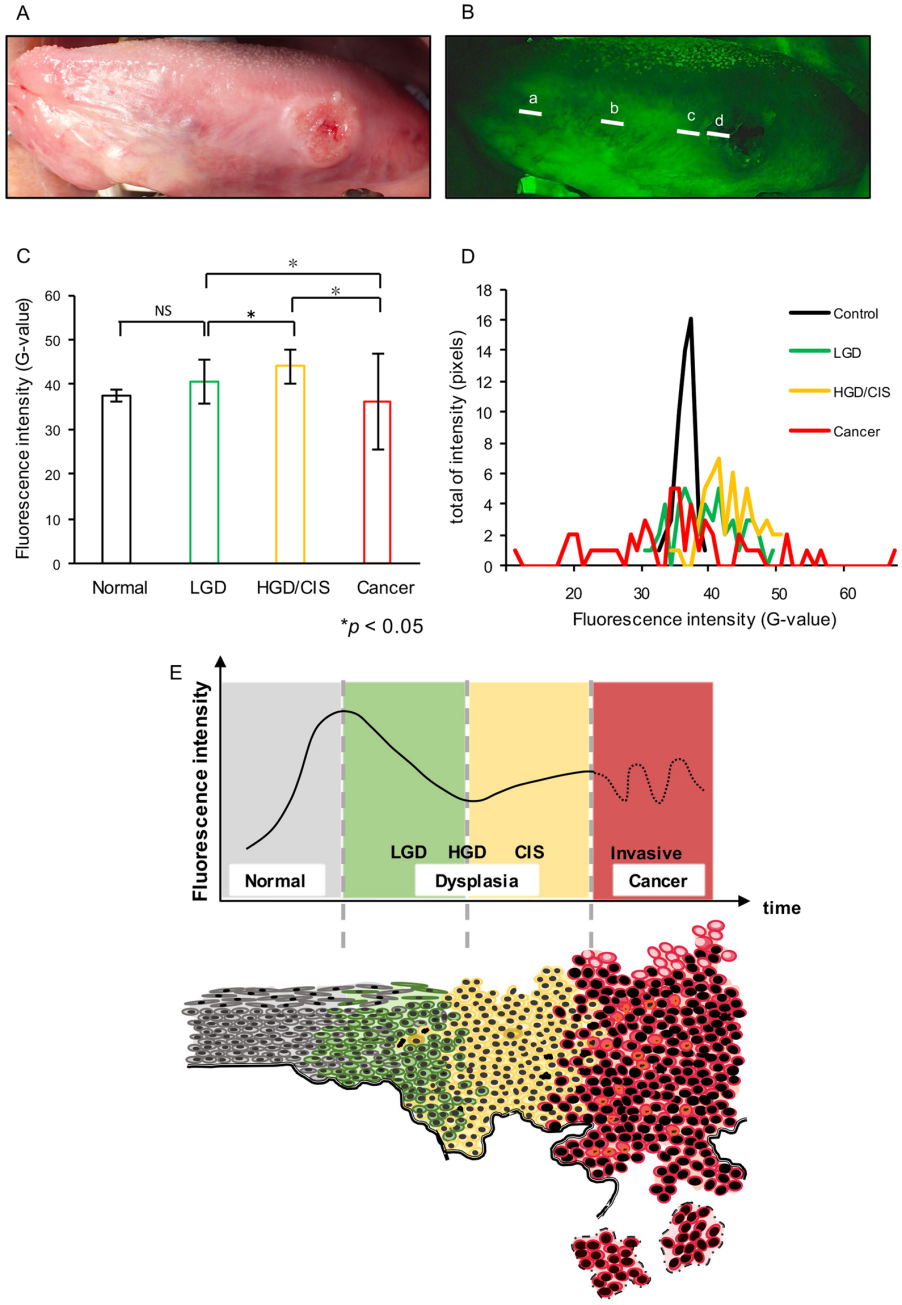
Practical application to human tongue cancer and relationship between the schema of tumor progression and fluorescence intensity transition. Gross observation (**A**) and fluorescence images (**B**) of a tongue cancer patient are shown. Measurement were taken at four sites (a: Control, b: low-grade dysplasia [LGD], c: high-grade dysplasia/carcinoma *in situ* [HGD/CIS], d: Cancer). The white line indicates 50 pixels. The mean fluorescence intensity (**C**) is shown, along with the scattering of fluorescence intensity (**D**). Significant differences were observed for all sites except between control and LGD (p < 0.05, Dunnett’s test). The transition of fluorescence intensity with the progression from normal epithelium to cancer (upper diagram) and tumor progression are illustrated (lower diagram) (**E**).

To prepare for the potential application of this method to evaluate human tongue cancer, gross observation and fluorescence imaging were performed with a human subject (Fig. 4A,B). Four measurement sites were chosen, including Normal (control), LGD, HGD/CIS and Cancer (Fig. 4B). The mean FI was recorded at these sites, and the scattering and distribution of FI was analyzed as in the animal model of cancer.

The tumor exhibited redness and swelling around the margin of ulceration, along with erythema and leukoplakia around the cancerous region (Fig. 4A). Fluorescence imaging showed a dark spot with a marbled pattern across the whole tumor area. The mean FI (G-values) were 37.4 (Normal), 40.6 (LGD), 44.1 (HGD/CIS), and 36.2 (Cancer) (Fig. 4C). The fluorescence of normal and LGD tissues were significantly lower in intensity than that of HGD/CIS. The FI was statistically significantly further reduced compared to other sites in the transition from HGD/CIS to Cancer. Regarding the scattering of each site, the Normal area exhibited little scattering and a narrow distribution, with G-values between 35 and 40. On the other hand, the distribution of G-values for LGD was 35 to 50, while HGD/CIS had a range of 30 to 50 and the Cancer region had the largest distribution of all groups, with G-values between 10 and 70 (Fig. 4D). These results showed that FI changes with progressing tumor malignancy, where increased scattering indicated greater tumor progression.

To measure the spread of the data distribution, the cut off was corrected to distinguish between the normal group and the lesion group using sample standard deviation of each group’s pixel value instead of mean G-values. In the rat model, we established that the sample standard deviation of Control was 2.43, LGD was 3.79, HGD/CIS was 3.68, and Cancer was 4.42. For humans, increased variability of FI was observed. The control was 1.34, the LGD was 4.72, the HGD/CIS was 3.74, and the cancer was 10.59. The widest variation was observed in the cancer stage. As a result, it was shown that the increase in fluctuation indicates a malignant tumor, which is similar to that observed in rats (Table 3).

**Table 3:** Variation of FI with tumor progression.

	Control	LGD	HGD/CIS	Ca
Animal model	2.43	3.79	3.68	4.42
Human	1.34	4.72	3.74	10.59

$${S}=\sqrt{\frac{1}{{N}-1}{\sum }_{{\boldsymbol{i}}=1}^{{\boldsymbol{N}}}{(xi-\bar{x})}^{2}}$$

S: Sample standard deviation, xi: One sample value, $\bar{{\rm{x}}}$: Sample mean, *N*: sample size.

## Discussion

In previous studies, FV diagnosis has been useful for the early detection of malignancies due to its simple and noninvasive application^21,22^. Previously there have been no consistent criteria, and it was simply used to identify dark areas. The appearance of dark spots has been attributed to decreasing autofluorescence from flavin adenine dinucleotide and collagen in abnormal epithelium^23^. The aim of this study was to statistically analyze FI and, through a noninvasive method, accurately evaluate it to detect and diagnose early-stage cancer under a consistent system of tumor progression. For this purpose, we established a 4NQO rat cancer model, which exhibits similar histology as human oral carcinogenesis^24,25^. Moreover, this animal model reliably progressed to squamous cell carcinoma. We discovered that 10, 15, and 20 weeks of 4NQO intake corresponded to LGD, HGD/CIS and Cancer, respectively. In addition, as a new method for evaluating mucosal lesions, we calculated the distribution (variation) and slope of the linear approximations using this reproducible animal model.

In this study, we found that the FI of normal tissue had uniformly low intensity, and thereafter FI significantly increased as the lesion progressed to LGD. This finding has not been found in previous observations. The increasing variation was caused as the regular epithelium underwent tumor progression, changing its reflection of fluorescence in the very early phase. With increasing 4NQO exposure, the epithelium changed to HGD/CIS and then cancer, resulting in FI with significantly lower intensity, Consistent with current knowledge. The tongue root area of this animal model exhibited malignant transformation more strongly than the rest of the tongue. Therefore, the “R” point (rear lesion of the tongue) had lower intensity than the “A” point (anterior lesion of tongue), and all abnormal experimental samples had negative slopes (Fig. 3C). In addition, the scattering of FI was also related to the increase in variation of FI accompanying tumor progression.

We hypothesize that the biggest cause for this variation is the condition of the epithelium. The normal epithelium has a thin keratin layer and uniform thickness, through which fluorescence could permeate, showing dark fluorescence due to weak reflection. No scattering was shown, owing to the uniform thickness of the normal epithelium. After the transition from normal to LGD stage epithelium, the FI was highly increased, because the epithelium of LGD developed a thick keratin layer with inflammation plus increased cell consistency, as the epithelial cells proliferated. The LGD surface therefore became a hard epithelium, resulting in strongly reflected FI and the emission of strong pale green fluorescence. This LGD epithelium was irregular and had mild scattering, but the FI was increased. We suggest that the reflection from FV was a stronger factor than the decrease in FI from mild scattering. In the HGD/CIS stage, the cell consistency was higher than in the LGD stage, with further altered keratin thickness and surface morphology. These changes led to additional scattering and decreased FI compared to the LGD stage, which manifested as an area of low intensity in the HGD/CIS stage. This decrease in FI with tumor progression has been reported in human patients as the same change in FI through the early stages to late stage cancer^26^. The epithelium was showed extensive scattering in the Cancer stage, appearing as dark spots like HGD/CIS. In addition, the FI of LGD, HGD/CIS, and Cancer stages was higher than that of the normal stage.

The emission of LGD appeared bright, while the HGD/CIS and Cancer stages appeared as dark surfaces. One suggested reason is that the LGD epithelium has increased keratin thickness, causing a hardening of its surface as an unavoidable consequence of the increased cell proliferation and inflammation associated with tumor progression. Lesions in the HGD/CIS and Cancer stages had lost uniformity and developed deep, rough surfaces. We suggest that FVL arises with tumor progression very early in premalignant inflammation, which is involved in the reduction and variability of FI as it progresses to more malignant stages (Fig. 4E).

Here, we investigated whether FI could be the cut off threshold in each stage. In case of control vs each lesion was shown high sensitivity and specificity. These cases could be set cut off around 38 (G-value). Likewise, LGD vs HGD or Cancer was observed that it could be set cut off around 50 (G-value). In other group including HGD and Cancer, it was low sensitively and specificity. The G-value cut off threshold results were similar to that observed in human. From “Normal to Carcinoma”, FI was increased from a low homogeneous state to a uniform state with LGD while maintaining a homogeneous condition, and decreased with the transition to HGD, and the progress to Carcinoma was expressed as an increase in variation. The FI of each of these distributions gradually changed over a wider range. In this research, we evaluated the case of one person as a pilot case. However, further analysis of data will increase accuracy. As a result, it may be possible to easily and accurately evaluate the progress from precancerous lesion to malignancy, which is difficult to evaluate, only by noninvasive observation.

Finally, we evaluated human tongue squamous cell carcinoma using this new analysis method. Comparing the *in vivo* and *ex vivo* measurements, we found that the Normal group had homogeneous variations. On the other hand, the lesion groups had a wide-range variation. These results suggested that this study was able to quantitatively differentiate between normal condition and dysplasia. However, evaluation of the *ex vivo* was adversely affected by many environmental factors such as natural light, brightness in the room, and dental prosthesis. To increase the effectiveness of FV, other reflections from these environments should be blocked.

Development of imaging technology in recent days has well known applications to not only FV device (IllumiScan^®^) but also near-infrared (NIR) fluorescence imaging. The FV device can transmit light to a tissue depth of 400–600 nm. Near-infrared (NIR) fluorescence imaging has relied on fluorophores that emit in the 700–900 nm NIR-I or 1,000–1,700 nm NIR-II window for generating deep-tissue images. Especially NIR-II makes it possible to achieve deep tissue imaging with a high spatial resolution owing to the lower autofluorescence and scattering of NIR light. For convenient and frequent examination, FV device is useful, and detailed depth analysis is useful for NIR. Therefore, depending on the case, it is considered effective to use in combination^27^. From the perspective of the future, we anticipate a new method for identifying malignancy by the color of reflection from autofluorescence.

## Conclusion

In conclusion, we demonstrated that FI migration and an uneven distribution are consistent with tumor progression. Since each step of tumor progression occurs reproducibly in this animal model, statistical evaluation was possible. This study demonstrated that the distribution of FI was increased with tumor progression, and our new analytical method might be useful for evaluating human tongue squamous cell carcinoma.

## Materials and Methods

### Animals

Male Sprague-Dawley (SD) rats (6 weeks old; weight, approximately 250 g; Sankyo Labo, Tokyo, Japan) were fed a standard diet. The animals were randomly divided into three groups, and were treated with 50 ppm 4NQO through their drinking water for 10, 15, and 20 weeks in 11, 15, and 14 animals, respectively. Ten non-treated rats were used as a control group (male SD rats, 10 weeks old; weight, approximately 250 g).

### Ethical statement

This research on animal subjects was approved by the Tokyo Dental College Review Board (Approval #290709). All animals received humane care in accordance with the guidelines for the treatment of experimental animals.

This research on human subjects was approved by the Tokyo Dental College Review Board (Approval #740). Written informed consent was obtained from each participant. All experiments were performed in accordance with relevant guidelines and regulations.

### Treatment with 4NQO

The 4NQO solution was made as previously reported. Briefly, a stock solution was prepared by dissolving 1.0 g of 4NQO (Sigma-Aldrich, USA) in 50 mL of ethanol and 4,950 mL of distilled water, which was stored at room temperature in the dark. The stock solution was diluted with tap water to a final concentration of 50 ppm, and light-resistant water bottles were filled with this diluted solution. Bottles were refilled with freshly prepared 4NQO solution once a week^19,20^.

### Experimental design

This experiment comprised three steps. The first step was to establish a 4NQO-induced rat tongue cancer model. In the second step, fluorescence images were recorded at each time period using FV (IllumiScan ® Shofu Inc, Kyoto, Japan) after rats were sacrificed evaluated histopathologically. In the third step, we measured FI using ImageJ version 1.51 and performed quantitative and statistical analyses (Fig. 1A).

### Gross observation and fluorography

Whole rat tongues were excised, and both white light and fluorography images were taken by FV to observe gross findings. For fluorography, each sample was placed in a black box to prevent light reflection and entry (Fig. 1B). The subject distance was 5 cm for both gross observation and fluorography (Fig. 1C,D). Each tongue was cut sagittally into two sections. The samples were fixed in 10% buffered formalin, embedded with paraffin, and then sliced into 5-μm thick sections using a microtome. The sections were stained with hematoxylin and eosin for histopathological analysis.

### Measurement site

In the rat model, the anterior part of the half-moon structure was the most suitable for evaluation because early lesions form there. The measured length corresponded to 200 pixels (Fig. 1D,E). We used ImageJ to measure FI and analyzed the surface plot, scattering of FI, and the linear approximations. We then performed statistical analysis of these quantitative data.

For human tongue cancer, FV data was measured at four sites: Normal (control), LGD, HGD/CIS, and Cancer, as determined from postoperative histological evaluation.

### Evaluation of the tongue

We evaluated the average FI value, the scattering (i.e., the total number of fluorescent pixels), and the inclination line. The inclination line, y = ax + b, was determined as the minimum distance from all measured FI values, and we evaluated the degree of inclination in each group.

### Statistical analysis

All data are presented as the mean ± standard deviation. For statistical analysis, non-repeated measures ANOVA, Dunnett’s test, Pearson correlation, and Sample standard deviation were used to compare control and experimental values. P values less than 0.05 were considered statistically significant.
